# A cross-sectional study of blood cultures and antibiotic use in patients admitted from the Emergency Department: missed opportunities for antimicrobial stewardship

**DOI:** 10.1186/s12879-016-1515-1

**Published:** 2016-04-18

**Authors:** Laura J. Shallcross, Nick Freemantle, Shasta Nisar, Daniel Ray

**Affiliations:** UCL Centre for Infectious Disease Informatics, Farr Institute of Health Informatics Research, 222 Euston Road, London, NW1 2DA UK; Department of Primary Care and Population Health, Upper Third floor, UCL Medical School, Rowland Hill Street, London, NW3 2PF UK; Health Informatics, University Hospitals Birmingham NHS Foundation Trust, Queen Elizabeth Hospital, Queen Elizabeth Medical Centre, Birmingham, B15 2TH UK

**Keywords:** Antimicrobial stewardship, Emergency Department, Health policy

## Abstract

**Background:**

Early review of antimicrobial prescribing decisions within 48 h is recommended to reduce the overall use of unnecessary antibiotics, and in particular the use of broad-spectrum antibiotics. When parenteral antibiotics are used, blood culture results provide valuable information to help decide whether to continue, alter or stop antibiotics at 48 h. The objective of this study was to investigate the frequency of parenteral antibiotic use, broad spectrum antibiotic use and use of blood cultures when parenteral antibiotics are initiated in patients admitted via the Emergency Department.

**Methods:**

We used electronic health records from patients admitted from the Emergency Department at University Hospital Birmingham in 2014.

**Results:**

Six percent (4562/72939) of patients attending the Emergency department and one-fifth (4357/19034) of those patients admitted to hospital were prescribed a parenteral antimicrobial. More than half of parenteral antibiotics used were either co-amoxiclav or piperacillin-tazobactam. Blood cultures were obtained in less than one-third of patients who were treated with a parenteral antibiotic.

**Conclusions:**

Parenteral antibiotics are frequently used in those admitted from the Emergency Department; they are usually broad spectrum and are usually initiated without first obtaining cultures. Blood cultures may have limited value to support prescribing review as part of antimicrobial stewardship initiatives.

## Background

Growing concern about the clinical and economic impact of antimicrobial resistance has led to a major focus on antimicrobial stewardship to reduce inappropriate antibiotic prescribing.^.^

Suboptimal antimicrobial prescribing is likely to be common in the Emergency Department (ED) [1], where junior clinicians are under pressure to initiate antibiotics promptly in patients who are unwell [2], time is constrained and there are large numbers of prescribers with rapid staff turnover [3]. Most patients in hospital are admitted via the ED, so prescribing in the ED sets up the patterns of antimicrobial use across the hospital. Yet the ED has not been the focus of antimicrobial stewardship initiatives [4], partly because those who prescribe in the ED are rarely responsible for reviewing their prescribing decisions.

The clinical decision to revise antimicrobial therapy is strongly linked to the availability of a microbiological diagnosis. But, in many patients with suspected bacterial infection specimens are not submitted for microbiological testing. Recent data from Acute Trusts suggests that fewer than half of antibiotic prescriptions are reviewed within 48 h [5]. We used electronic health records (EHRs) to investigate the extent to which blood cultures are used when parenteral antibiotics are initiated in secondary care using data from University Hospitals Birmingham NHS Foundation Trust (UHB), the largest single-site hospital in England.

## Methods

We included data from UHB, a unique data holding which covers adult patient attendances at Queen Elizabeth Hospital Birmingham (QEHB). QEHB provides direct clinical services to nearly 800,000 adult patients per year and is a regional centre for specialist services including liver transplantation and renal dialysis. The local population is comparatively young and ethnically diverse, and includes patients with high levels of social deprivation. UHB captures large amounts of data from its patients in a number of different systems electronically, which can be linked together to evaluate patient status and clinical outcomes including information on diagnoses, treatments, investigations, test results and prescriptions.

To calculate the prevalence of parenteral antimicrobial use and blood culture sampling amongst patients attending the ED, and to investigate the clinical and microbiological outcomes for those patients who were admitted, we extracted the most recent year of data for a cohort of patients who attended the ED in 2014. We defined blood culture sampling as an electronic request for a blood culture recorded within 48 h following attendance at the ED. Use of parenteral antibiotics was defined as a prescription for a parenteral antibiotic within 48 h of ED attendance. This 48 h time period was chosen because blood cultures requested overnight may not be registered on the database until the following morning, potentially underestimating the frequency of blood culture use in this setting. The admission diagnosis was derived from the primary ICD-code for the whole admission.

## Results

**Fig. 1:**
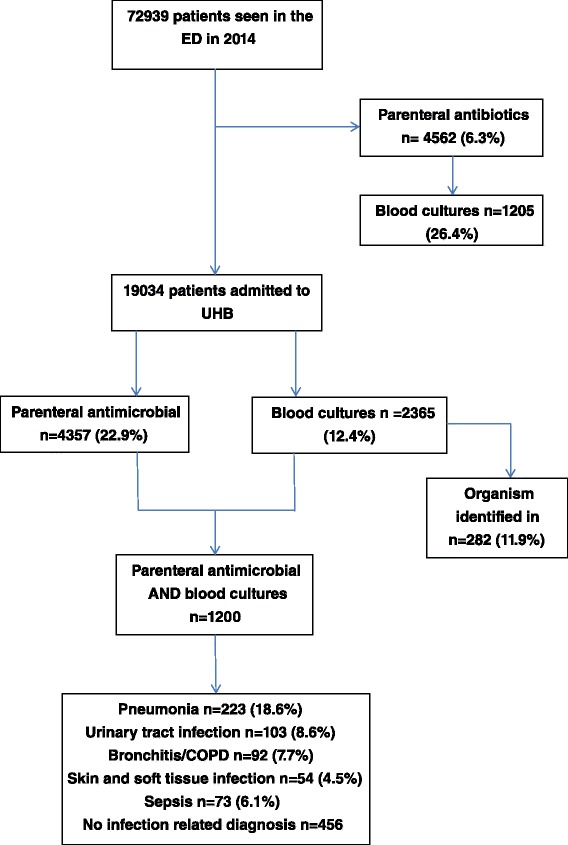
The use of parenteral antibiotics and blood cultures in patients attending the Emergency Department

In 2014, 72939 patients were seen in the ED at QEHB, representing 106,119 attendances, Fig. 1. At least one parenteral antimicrobial was prescribed for 4562 (6.3 %) of these patients within the following 48 h, most of whom did not have a blood culture taken (3347/4562; 73.3 %). 1300 patients had a blood culture taken but were not prescribed an antimicrobial over the same time period.

**Fig. 2:**
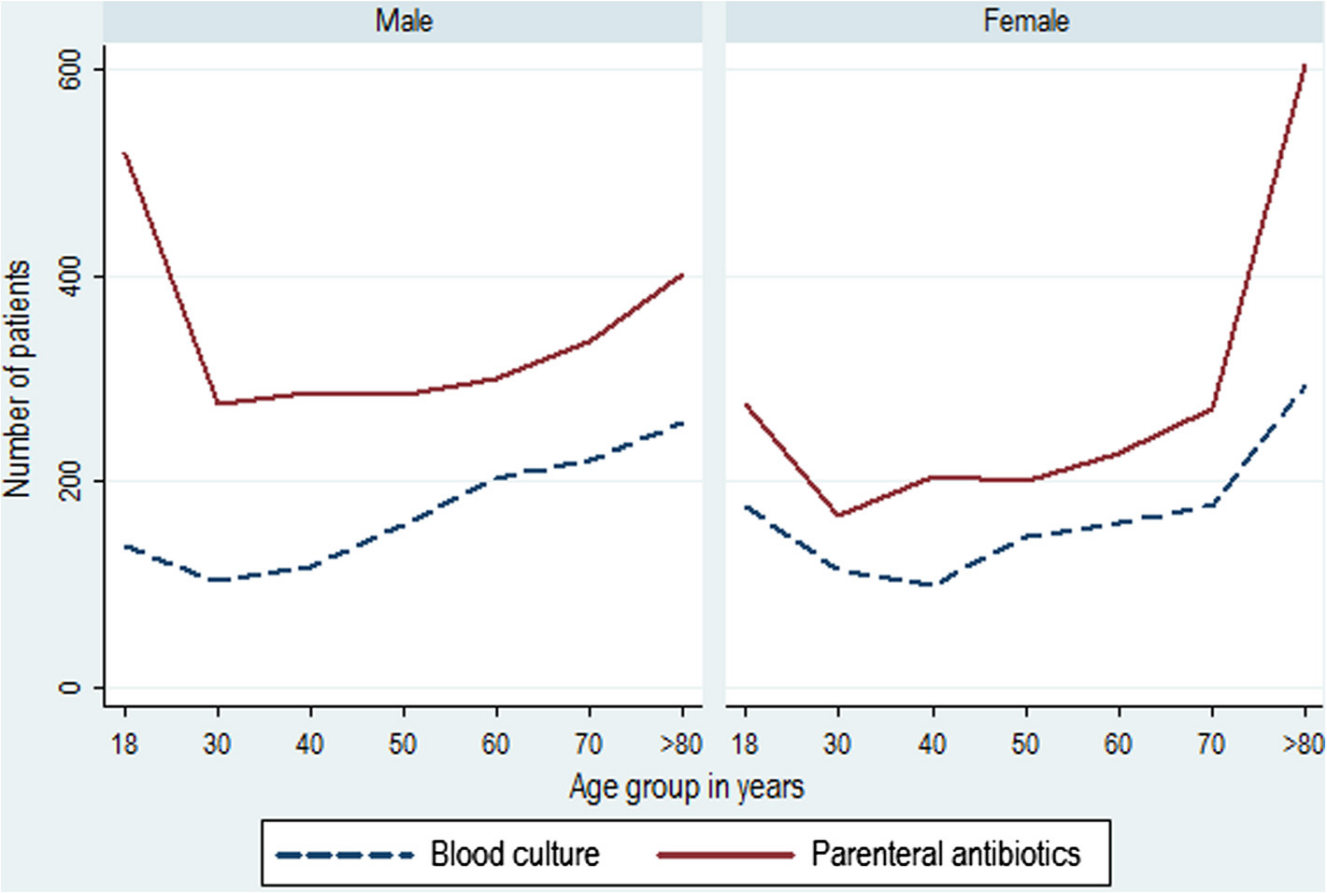
Use of blood cultures and parenteral antibiotics within the 48 h following hospital admission, by age and gender, 2014

Amongst 19034 patients who were admitted to hospital from the ED, 4357 (22.9 %) received a parenteral antimicrobial within the first 48 h and 2365 patients (12.4 %) had a specimen taken for blood culture, Fig. 1. An organism was identified in 11.9 % (282/2365) of patients with a blood culture, with *E. coli* (78/282) and *S. aureus* (21/282) as the leading causes of bacteraemia, Table 1. Overall the use of blood cultures and parenteral antibiotics was more frequent in men compared to women and increased from age 30 years and above, rising sharply in the elderly, from age 60 years in men and age 70 years in women. At all ages the use of parenteral antibiotics far exceeded the use of blood cultures, Fig. 2.

**Table 1:** Organism growth from blood cultures sampled from patients admitted to UHB in 2014

Organism	Number of patients (%)
*E. coli*	78 (27.7)
*Coagulase negative Staphylococcus*	44 (15.6)
*Staphylococcus aureus*	21 (7.4)
*Klebsiella pneumonia*	10 (3.5)
*Streptococcus pneumonia*	13 (4.6)
*Beta haemolytic streptococcus group A*	6 (2.1)
*Proteus mirabilis*	8 (2.8)
*Corynebacterium sp.*	7 (2.5)
Other	95 (33.7)
Total	282 (100)

Data on diagnosis was available for 1200 patients who had both a blood culture taken and were prescribed a parenteral antibiotic within 48 h following emergency admission to hospital. Pneumonia was the most common diagnosis amongst this patient group (18.6 %), followed by urinary tract infection (8.6 %), an exacerbation of COPD (7.7 %), skin and soft tissue infection (5.6 %) and sepsis (6.1 %). For at least 456 (38 %) of this group an infection-related diagnosis was not recorded as the primary reason for admission.

Data on the type of antimicrobial were available for 4474 patients prescribed a parenteral antibiotic within the 48 h following emergency admission to hospital. Co-amoxiclav was most the most commonly prescribed antibiotic (29.9 %), followed by piperacillin-tazobactam (20.0 %), flucloxacillin (16.0 %), clarithromycin (8.2 %) and meropenem, which was prescribed to one in twenty patients.

## Discussion

In this study using real-world data from the largest single site hospital in England, parenteral antimicrobials were prescribed to six percent of patients attending the ED and 23 % of patients admitted to hospital. Blood cultures were obtained from less than one-third of patients who were treated with parenteral antimicrobials at emergency admission to hospital and a micro-organism was identified in just 12 % of patients who had a blood culture. More than half of all patients prescribed a parenteral antimicrobial were treated with the broad spectrum antibiotics co-amoxiclav or piperacillin-tazobactam.

Although the rate of pathogen detection by blood culture was low in our study, it is comparable to other studies conducted in the ED [6, 7]. There are many factors that can reduce the sensitivity of blood cultures such as collection of a low volume sample, poor sampling technique and prior treatment with antimicrobials, and a further problem is the rate of false positive tests through blood culture contamination [8]. We were unable to determine the proportion of blood cultures that were obtained after antimicrobial treatment had commenced.

A key recommendation in antimicrobial stewardship guidance is to obtain microbial cultures before starting antibiotic treatment, provided it does not delay treatment for patients with sepsis or severe infection [9]. National guidance recommends that blood cultures are obtained from all patients with moderate to severe community onset pneumonia [10], the major cause of infection in our cohort. Guidance on the use of blood cultures in patients presenting with urinary tract infection or skin and soft tissue disease is less clear, but there is consensus that blood cultures should be obtained from patients with suspected sepsis [11].

Our research suggests blood cultures are obtained from less than one-third of patients who are initiated on parenteral antibiotics at admission to hospital. For a subset of patients without a blood culture, specimens are likely to have been obtained from the site of local infection (urine, wound swabs, sputum). Nonetheless there will be a substantial proportion of patients who are treated with parenteral antibiotics for whom is no microbiological specimen diagnosis. This presents a challenge for clinicians at prescribing review because they are required to make a decision about the need for ongoing antimicrobial treatment without a microbiological diagnosis, using clinical assessment and investigations.

Whilst the majority of clinicians acknowledge that antimicrobials are overused in hospital, it is extremely challenging to change prescribing behaviour in the absence of sensitive and specific diagnostic tests that can reliably rule out bacterial infection. More than half of the antibiotics prescribed to patients in this study were broad spectrum, despite increasing evidence that broad-spectrum prescribing is associated with selection of antimicrobial resistance bacteria, including selection of extended-spectrum beta-lactamase (ESBL) producing gram-negative bacteria and Methicillin-resistant *Staphylococcus aureus* (MRSA) [12, 13]. In a recent review [14], four heterogeneous studies of interventions involving prescribing review all demonstrated a substantial improvement in prescribing quality [15–18]. However, only two of these studies focused on discontinuation of unnecessary antibiotics, either by increasing the role of infectious disease specialists to scrutinise antimicrobial prescribing [15], or by introducing a clinical scoring system to reduce unnecessary antibiotic use [16]. Clinical decision support systems can improve prescribing quality [19], but have yet to make a substantial impact on total antimicrobial use [20], whereas introducing pro-calcitonin to improve diagnostic accuracy for bacterial infection has been demonstrated to reduce the duration of antibiotic treatment in secondary care [21].

The strength of this study is that is reflects real clinical practice because QEHB is a paperless hospital and all patient activity is recorded electronically. The limitations are that our results may not be generalizable to other sites, particularly because QEHB has a unique informatics infrastructure. We analysed patient records at aggregate rather than individual-level, and were therefore unable to investigate clinical outcome in detail. We also restricted our analysis to prescriptions for parenteral antimicrobials, although the impact of excluding oral antimicrobials is likely to be small because patients who are unwell enough to be admitted to hospital are frequently commenced on parenteral antimicrobials. We only considered blood cultures and did not include other types of microbial samples.

## Conclusion

In patients admitted via the Emergency Department, blood cultures may have a limited role in the diagnosis of bacterial infection. Decisions to review antimicrobial prescribing should combine clinical assessment with a range of diagnostic information which includes, but is not limited to, culture-based microbiology.

## Ethics approval and consent to participate

This study was registered as an audit with University Hospitals Birmingham (ref CARMS-11965). Full ethical approval was not required because all analyses were undertaken on aggregated and fully anonymised datasets as set out by the Health Research Authority (http://www.hra.nhs.uk/resources/before-you-apply/research-requiring-nhs-rd-review-but-not-ethical-review/). Permission to access the dataset was provided by the data guardian at UHB (D. Ray).

## Availability of data and materials

NHS data analysed in this study cannot be publicly shared because data access is granted on a named –individual basis.

## Abbreviations

E. coli: *Escherichia coli*
ED: emergency department
EHR: electronic health records
ESBL: extended spectrum beta-lactamase
ICD: international classification of diseases
MRSA: methicillin resistant *Staphylococcus aureus*
QEHB: Queen Elizabeth Hospital Birmingham
*S. aureus*: *Staphylococcus aureus*
UHB: University Hospital Birmingham
